# Supplementary material for: Spin decoherence in a two-qubit CPHASE gate: the critical role of tunneling noise
Source: npj Quantum Inf. Author manuscript; Available in PMC 2024 Oct 23. (PMC11497488; doi:10.1038/s41534-018-0112-0)
Supplement: Electronic supplementary material [file NIHMS1526038-supplement-Electronic_supplementary_material.pdf]

Supplementary information of "Spin decoherence in a two-qubit logic gate: the critical role of tunneling noise"

Peihao Huang, Neil M. Zimmerman, and Garnett W. Bryant
(Dated: October 24, 2018)

S1. EFFECTIVE HAMILTONIAN

Here, we show a detailed derivation of the effective two-qubit Hamiltonian in the main text. We write $H = H_0 + H_1$, where H_0 is the Hamiltonian without noise and H_1 contains the noise terms. H_0 in the basis $|(1,1)T_+\rangle$, $|(1,1)T_0\rangle$, $|(1,1)S\rangle$, $|(1,1)T_-\rangle$, and $|(2,0)S\rangle$ is

$$H_0 = \begin{bmatrix} \overline{E}_Z & 0 & 0 & 0 & 0 \\ 0 & 0 & \frac{\delta E_Z}{2} & 0 & 0 \\ 0 & \frac{\delta E_Z}{2} & 0 & 0 & \sqrt{2}t_0 \\ 0 & 0 & 0 & -\overline{E}_Z & 0 \\ 0 & 0 & \sqrt{2}t_0 & 0 & U-\epsilon_0 \end{bmatrix}. \quad (1)$$

Figure S1 shows the energy diagram of the system as a function of the detuning of the DQD obtained by numerically diagonalizing H_0. A large value $\delta E_z = 2$ GHz (or 8.27 μeV) is used to ensure a visible splitting between $|\uparrow\downarrow\rangle$ and $|\downarrow\uparrow\rangle$. The other parameters are the same as in the main text. The two triplet states $|(1,1)T_+\rangle$ and $|(1,1)T_-\rangle$ are decoupled from the other three states as required by the Hamiltonian. As the detuning ϵ_0 becomes comparable to the onsite Coulomb energy U, admixture of the singlet states occurs, where strong spin decoherence can occur due to the admixture and charge noise.

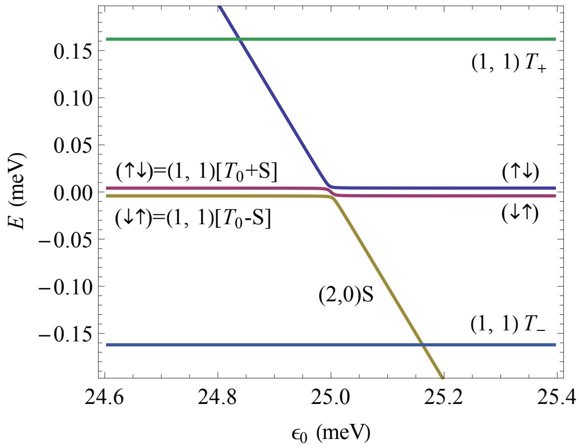

FIG. S1. Energy diagram (schematic) of the system versus the detuning of DQD. Admixture of singlet states occurs when the detuning ϵ_0 becomes comparable to $U = 25$ meV.

To study spin decoherence, it is convenient to find an effective Hamiltonian. In the limit $t_0 \ll U - \epsilon_0$, we can first eliminate the hopping term in H_0 by a rotation in the subspace of state $|(1,1)S\rangle$ and $|(2,0)S\rangle$. This results in an effective exchange interaction between electron spins. We then diagonalize H_0 through a second rotation, which results in an effective Hamiltonian. For simplicity, $|\alpha\rangle$ with $\alpha = 1, \cdots, 5$ is also used to represents each basis state.

In the subspace spanned by $|(1,1)S\rangle$ and $|(2,0)S\rangle$, the system Hamiltonian is

$$\begin{aligned} H = &-\Omega_{35}(\sigma_z \cos\theta_1 + \sigma_x \sin\theta_1) + \\ &\sqrt{2}\hat{n}_t\sigma_x - \hat{n}_\epsilon(1-\sigma_z)/2 + const., \end{aligned} \quad (2)$$

where σ_x and σ_z are the Pauli matrices defined in the subspace of $|3\rangle$ and $|5\rangle$, $\Omega_{35} = \sqrt{8t^2 + (U-\epsilon_0)^2}/2$, $\theta_1 = \tan^{-1}[-2\sqrt{2}t_0/(U-\epsilon_0)]$, and $const. = (U-\epsilon_0)/2$. The H_0 part can be diagonalized as

$$H_0' = -\Omega_{35}\sigma_z + const. \quad (3)$$

by a rotation $H' = R_y^\dagger H R_y$, where $R_y = \exp(-i\sigma_y\theta_1/2)$ rotates $|(1,1)S\rangle$ and $|(2,0)S\rangle$ to a new basis $|(1,1)S'\rangle$ and $|(2,0)S'\rangle$ (note that, in the limit of $t_0 \ll U - \epsilon_0$, θ_1 is reduced to θ in the main text, and the state $|(1,1)S'\rangle$ is approximately $|(1,1)S\rangle$ with a small admixture from $|(2,0)S\rangle$).

In the subspace spanned by $|(1,1)T_0\rangle$, $|(1,1)S'\rangle$ and $|(2,0)S'\rangle$, the effective Hamiltonian H' is

$$H' \approx \begin{bmatrix} 0 & \frac{\delta E_Z}{2}\cos\frac{\theta_1}{2} & \frac{\delta E_Z}{2}\sin\frac{\theta_1}{2} \\ \frac{\delta E_Z}{2}\cos\frac{\theta_1}{2} & J + \hat{n}'_{33} & \hat{n}'_{35} \\ \frac{\delta E_Z}{2}\sin\frac{\theta_1}{2} & (\hat{n}'_{35})^\dagger & U'_{55} + \hat{n}'_{55} \end{bmatrix}, \quad (4)$$

where the effective exchange interaction $J = (U-\epsilon_0)/2 - \Omega_{35} \approx -2t^2/(U-\epsilon_0)$, $U'_{55} = U - \epsilon_0 - J$ is the effective energy of double occupation state. The noise terms are $\hat{n}'_{33} = \sqrt{2}\hat{n}_t \sin\theta_1 - \hat{n}_\epsilon(1-\cos\theta_1)/2$, $\hat{n}'_{35} = \sqrt{2}\hat{n}_t\cos\theta_1 - \hat{n}_\epsilon(\sin\theta_1)/2$, and $\hat{n}'_{55} = -\sqrt{2}\hat{n}_t\sin\theta_1 - \hat{n}_\epsilon(1+\cos\theta_1)/2$. In the limit of $t_0 \ll U-\epsilon_0$, where $|\theta_1| \approx |-2\sqrt{2}t_0/(U-\epsilon_0)| \ll 1$, we have $\hat{n}'_{33} \approx \sqrt{2}\hat{n}_t\theta_1 - \hat{n}_\epsilon\theta_1^2/4$, $\hat{n}'_{35} = \sqrt{2}\hat{n}_t - \hat{n}_\epsilon\theta_1/2$, and $\hat{n}'_{55} = -\sqrt{2}\hat{n}_t\theta_1 - \hat{n}_\epsilon$.

Note that, in the limit of $|\theta_1| \approx |-2\sqrt{2}t_0/(U-\epsilon_0)| \ll 1$, the admixture of $|(1,1)T_0\rangle$ and $|(2,0)S'\rangle$ is negligible since $H'_{25}/U'_{55} \approx \theta_1\delta E_Z/U'_{55}/4$ is negligible. Therefore, state $|(2,0)S'\rangle$ is decoupled from $|(1,1)T_0\rangle$, and we have $H'_{25} \approx 0$. Similarly, to 1st order in θ_1, we have $H'_{23} \approx \delta E_Z/2$.

The H_0' part of Hamiltonian H' can further be diagonalized by a rotation in the subspace spanned by $|(1,1)T_0\rangle$ and $|(1,1)S'\rangle$. The resulting Hamiltonian in the basis of$|(1,1)T_+\rangle$, $|(1,1)T_0''\rangle$, $|(1,1)S''\rangle$, $|(1,1)T_-\rangle$, and

$|(2,0)S'\rangle$ is given by

$$H'' = \begin{bmatrix} \overline{E}_Z & 0 & 0 & 0 & 0 \\ 0 & U''_{22} + \hat{n}''_{22} & \hat{n}''_{23} & 0 & \hat{n}''_{25} \\ 0 & (\hat{n}''_{23})^\dagger & U''_{33} + \hat{n}''_{33} & 0 & \hat{n}''_{35} \\ 0 & 0 & 0 & -\overline{E}_Z & 0 \\ 0 & (\hat{n}''_{25})^\dagger & (\hat{n}''_{35})^\dagger & 0 & U'_{55} + \hat{n}'_{55} \end{bmatrix},$$

where $U''_{22} = J/2 + \Omega_J/2$, $U''_{33} = J/2 - \Omega_J/2$, and $\Omega_J = \sqrt{J^2 + \delta E_Z^2}$ is the energy splitting of state $|2''\rangle$ and state $|3''\rangle$. The angle θ_2 of rotation that diagonalizes H'_0 in the subspace $|2\rangle$ and $|3'\rangle$ satisfies $\cos\theta_2 = -J/\Omega_J$, and $\sin\theta_2 = \delta E_Z/\Omega_J$. The noise terms are $\hat{n}''_{22} = \hat{n}'_{33}(1 - \cos\theta_2)/2$, $\hat{n}''_{23} = \hat{n}'_{33}\sin\theta_2/2$, $\hat{n}''_{33} = \hat{n}'_{33}(1 + \cos\theta_2)/2$, $\hat{n}''_{25} = -\hat{n}'_{35}\sin(\theta_2/2)$, and $\hat{n}''_{35} = \hat{n}'_{35}\cos(\theta_2/2)$. Note that $\hat{n}''_{22}$, $\hat{n}''_{23}$ and $\hat{n}''_{33}$ are related to $\hat{n}'_{33}$, while $\hat{n}''_{25}$ and $\hat{n}''_{35}$ are related to $\hat{n}'_{35}$. In the two-qubit gate experiment, condition $J \ll \delta E_Z$ is satisfied. In this case, we have $\theta_2 \approx \pi/2$, $\hat{n}''_{22} \approx \hat{n}''_{23} \approx \hat{n}''_{33} \approx \hat{n}'_{33}/2$, $\hat{n}''_{25} = -\hat{n}''_{35} = -\sqrt{2}\hat{n}'_{35}/2$, $|2''\rangle = |\uparrow\downarrow''\rangle \approx (|(1,1)T_0\rangle + |(1,1)S'\rangle)/\sqrt{2}$, and $|3''\rangle = |\downarrow\uparrow''\rangle \approx (|(1,1)T_0\rangle - |(1,1)S'\rangle)/\sqrt{2}$.

S2. EFFECTIVE NOISE SPECTRUM $J^{(zz)}_{3''4}(\omega)$

In this section, we estimate the magnitudes of the detuning and tunneling fluctuations due to the presence of charge noise (see Figure S2), and then derive the effective noise spectrum $J^{(zz)}_{3''4}(\omega)$. We also discuss the possibility of correlated tunneling and detuning noise.

The detuning is the energy difference of electron chemical potential in the left and right QD. If we suppose that the charge noise fluctuations at the left and right QD are not correlated, then, the detuning fluctuation $\delta\epsilon$ should be on the same order of the chemical potential fluctuation $\delta\mu$ in a single QD, i.e.

$$\delta\epsilon \approx \delta\mu. \tag{5}$$

To estimate the amplitude of tunneling fluctuations due to charge noise, we consider $t_0 \approx E_0 e^{-l_b\sqrt{2m^*(E_b - E_0)/\hbar^2}}$ according to the WKB approximation [1, 2], where E_0 is the orbital energy of a single QD, E_b and l_b are the effective barrier height and width. Since the charge noise is believed to be from noise-producing defects homogeneously distributed in the plane of the device, the magnitude of charge noise should be quite uniform along the interface. Therefore, we assume that the fluctuation of tunnel barrier δE_b due to charge noise is on the same order of chemical potential fluctuations $\delta\mu$ in a single QD; then, the tunneling fluctuation δt_0 due to charge noise is

$$\delta t_0 \approx (\partial t_0/\partial E_b)\delta\mu, \tag{6}$$

where the ratio $\partial t_0/\partial E_b \approx t_0/(2\Delta_b)$ converts the barrier fluctuation to the fluctuation of tunneling rate and

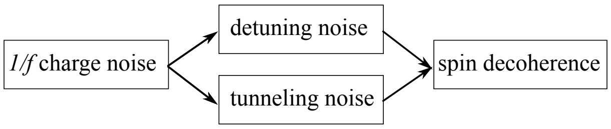

FIG. S2. Effect of $1/f$ charge noise on spin decoherence in a two-qubit logic gate.

$\Delta_b \equiv \sqrt{(E_b - E_0)\hbar^2/(2m^* l_b^2)}$. Suppose $E_0 = 3$ meV, $l_b = 20$ nm, $E_b = 20$ meV, we have $\Delta_b \approx 3$ meV. Then, $\delta t \approx 4\times 10^{-3}\delta\mu$. We should emphasize that, although the WKB approximation is not an accurate method to evaluate the tunneling rate, it is a convenient way to give a rough qualitative estimate. The actual value of conversion ratio $\delta t/\delta\mu$ could be different from $t_0/(2\Delta_b)$; however we believe the value is not off by orders of magnitude, and the small variations will not affect our main results.

From Eq. (7) in the main text, we have

$$J^{(zz)}_{3''4}(\omega) = \frac{2}{\hbar^2}\int_{-\infty}^{\infty} \langle \hat{h}^{(z)}_{3''4}(0)\hat{h}^{(z)}_{3''4}(\tau)\rangle \cos(\omega\tau)d\tau,$$

where $\hat{h}^{(z)}_{3''4} = \hat{n}''_{33}/2 = \hat{n}'_{33}/4 = \frac{1}{4}[\sqrt{2}\theta\hat{n}_t - (\theta^2/4)\hat{n}_\epsilon]$.

Note that, the magnitude of tunneling noise $\hat{n}_t$ is related to the magnitude of detuning noise $\hat{n}_t \approx (\partial t_0/\partial E_b)\hat{n}_\epsilon$; however, the tunneling noise and the detuning noise may not be fully correlated. The correlation between the two noises can be characterized by a parameter r,

$$\langle \hat{n}_t(0)\hat{n}_\epsilon(0)\rangle = r\frac{\partial t_0}{\partial E_b}\langle \hat{n}_\epsilon(0)\hat{n}_\epsilon(\tau)\rangle, \tag{7}$$

where $0 \le r \le 1$. When $r = 0$, the two noise is uncorrelated; when $r = 1$, the two noises are fully correlated; when $0 < r < 1$, the noises are partially correlated. We expect that the noises are in general uncorrelated, $r = 0$, due to the fact that the two types of noise are from different events at different locations. The fluctuation of detuning is due to the noise at each QD, while the fluctuation of tunneling is considered to be a result of events at the location of tunnel barrier.

We expect that the noises are in general uncorrelated, $r = 0$, due to the fact that the two types of noise are from different events at different locations. To support this, we first observe that strongly correlated noise could arise from two mechanisms: i) single defects could strongly affect both the tunnel barrier and the quantum dot chemical potential; ii) many defects distributed in different locations could have correlated fluctuations. Regarding the first possibility, if one considers the cross capacitances [3], the fluctuation of a single defect will not couple significantly to both the tunnel barrier and the quantum dot. Regarding the second possibility, defect fluctuations are generally not correlated with each other, since strain fields do not extend over long distances (the

typical distance between tunnel barrier and quantum dot is of order tens of nm).

In the following, we discuss the spectral density for uncorrelated, fully correlated, and partially correlated noises.

When $r = 0$, the noises are uncorrelated, we have,

$$\begin{aligned} J^{(zz)}_{3''4}(\omega) &= \frac{2}{16\hbar^2}\int_{-\infty}^{\infty} 2\theta^2\langle\hat{n}_t(0)\hat{n}_t(\tau)\rangle\cos(\omega\tau)d\tau, \\ &+ \frac{2}{16\hbar^2}\int_{-\infty}^{\infty}\frac{\theta^4}{16}\langle\hat{n}_\epsilon(0)\hat{n}_\epsilon(\tau)\rangle\cos(\omega\tau)d\tau, \\ &= \frac{1}{8\hbar^2}\left[2\theta^2 S_{t_0}(\omega) + \frac{\theta^4}{16}S_\epsilon(\omega)\right], \end{aligned} \tag{8}$$

since $\delta\epsilon \approx \delta\mu$, we have

$$S_\epsilon(\omega) = S_{1/f}(\omega) = A/\omega, \tag{9}$$

$$S_{t_0}(\omega) = (\partial t_0/\partial E_b)^2 A/\omega. \tag{10}$$

Therefore, the effective noise spectral density is

$$J^{(zz)}_{3''4}(\omega) = A_{eff}/\omega, \tag{11}$$

where $A_{eff} = \frac{1}{8\hbar^2}[2\theta^2(\partial t_0/\partial E_b)^2 + \theta^4/16]A$.

When $r = 1$, the noises are fully correlated, we have,

$$J^{(zz)}_{3''4}(\omega) = \frac{1}{16}\left[\sqrt{2}\theta\frac{\partial t_0}{\partial E_b} - \frac{\theta^2}{4}\right]^2\frac{2}{\hbar^2}S_\epsilon(\omega), \tag{12}$$

Therefore, the effective noise spectral density is

$$J^{(zz)}_{3''4}(\omega) = A_{eff}/\omega, \tag{13}$$

where $A_{eff} = \frac{1}{8\hbar^2}[\sqrt{2}\theta(\partial t_0/\partial E_b) + \theta^2/4]^2 A$.

When $0 < r < 1$, the noises are partially correlated, we have,

$$\begin{aligned} J^{(zz)}_{3''4}(\omega) = \frac{1}{8\hbar^2}\Bigg[& 2\theta^2 S_{t_0}(\omega) + \frac{\theta^4}{16}S_\epsilon(\omega) \\ & - \frac{\sqrt{2}}{2}\theta^3 r\frac{\partial t_0}{\partial E_b}S_\epsilon(\omega)\Bigg], \end{aligned} \tag{14}$$

Therefore, the effective noise spectral density is

$$J^{(zz)}_{3''4}(\omega) = A_{eff}/\omega, \tag{15}$$

where $A_{eff} = \frac{1}{8\hbar^2}[2\theta^2(\partial t_0/\partial E_b)^2 - \theta^3 r(\partial t_0/\partial E_b)/\sqrt{2} + \theta^4/16]A$.

Regardless of the value of r, the relative contributions from tunneling and detuning noise depend on the relative strength.

S3. ANALYTIC EXPRESSION OF DEPHASING DYNAMICS

Here we analyze the dephasing dynamics. The system dephases as $\exp[-\phi(t)]$, where the phase factor is

$$\phi(t) = \int_{\omega_L}^{\omega_U} d\omega J^{(zz)}_{\alpha\beta}(\omega)[2\sin(\omega t/2)/\omega]^2, \tag{16}$$

where ω_L and ω_U are the lower and upper limit of frequency, ω_L represents the time scale of an overall measurement of coherent dynamics, and ω_U represents the shortest times scale of taking a single shot measurement in experiment. Therefore,

$$\phi(t) = A_{eff}\int_{\omega_L}^{\omega_U} d\omega\frac{4\sin^2(\omega t/2)}{\omega^3} = A_{eff}t^2 f(x)\Big|_{x_L}^{x_U},$$

where $x_L = \omega_L t$, $x_U = \omega_U t$,

$$f(x) = \int dx\frac{4\sin^2(x/2)}{x^3}. \tag{17}$$

One finds that

$$f(x) = \frac{\cos x - 1}{x^2} - \frac{\sin x}{x} + Ci(x), \tag{18}$$

where $Ci(x)$ is the cosine integral function.

In most experiments, we have $x_L = \omega_L t \ll 1$ and $x_U = \omega_U t \gg 1$. We can expand $f(x_L)$ in the limit of $x_L \ll 1$ and $f(x_U)$ in the limit of $1/x_U \ll 1$. When $x \ll 1$, we have

$$Ci(x) \approx \gamma + \ln(x) - x^2/4,$$

where $\gamma \approx 0.577216$ is the Euler constant. Thus,

$$f(x_L) \approx -3/2 + \gamma + \ln(x_L) - x_L^2/24. \tag{19}$$

When $x \gg 1$, we have

$$Ci(x) \approx \frac{1}{x}\sin(x) - \frac{1}{x^2}\cos x.$$

Thus,

$$f(x_U) \approx \frac{1}{x_U^2}\left[-1 + \frac{6}{x_U^2}\cos x_U - \frac{2}{x_U}\sin x_U\right]. \tag{20}$$

Therefore, the dynamics of $\phi(t)$ is given by

$$\phi(t) \approx A_{eff}t^2\left[\frac{3}{2} - \gamma - \ln(x_L)\right] \approx -A_{eff}t^2\ln(\omega_L t), \tag{21}$$

where the logarithmic function $\ln(\omega_L t)$ modifies coherence dynamics from $e^{-(t/T_\varphi)^2}$ to a slower time dependence. The logarithmic dependence is in contrast with the results in Ref. [4] since we are interested in the long time limit of dephasing dynamics, where $x_U = \omega_U T_\varphi \gg 1$ is satisfied.

S4. DEPHASING DYNAMICS

Figure S3 left panel shows the time dependence of spin dephasing dynamics $\exp[-\phi(t)]$ of the TQ in the presence of tunneling noise for various detuning ϵ_0 (C-Q is spin down). The magnitude of $1/f$ charge noise $A = (2\mu eV)^2$ and $\partial t_0/\partial E_b = 10^{-3}$. The qubit dephasing

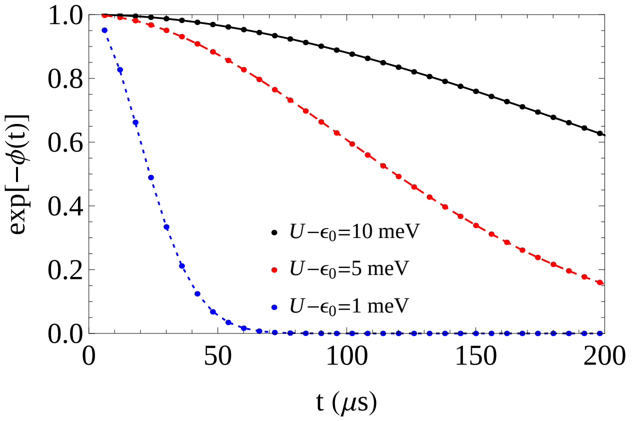

FIG. S3. Dephasing dynamics $\exp[-\phi(t)]$ as a function of time for detuning $\epsilon_0 = 15$, 20, and 25 meV in the presence of tunneling noise with $A = (2\mu eV)^2$ and $\partial t_0/\partial E_b = 10^{-3}$. The dots are numerical results, which are fitted to $e^{-(t/T_\varphi)^\beta}$ (colored lines) with $\beta \approx 1.90$. Spin coherence decays faster as ϵ_0 get close to U.

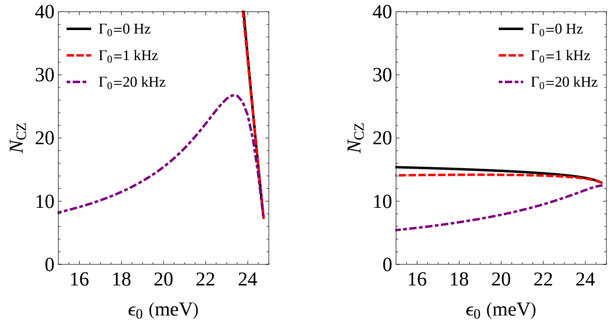

FIG. S4. Left: N_{CZ} as a function of detuning ϵ_0 due to detuning noise only, where $A = (2\mu eV)^2$. Right: Same plot for tunneling noise, where $A = (2\mu eV)^2$ and $\partial t_0/\partial E_b = 3.2 \times 10^{-3}$. When tunneling noise dominates, N_{CZ} increases as ϵ_0 approaches U (more asymmetry) in the presence of a finite Γ_0.

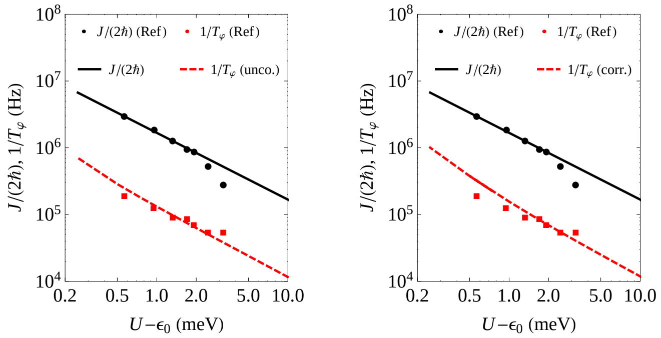

FIG. S5. Left: $J/(2\hbar)$ and $1/T_\varphi$ as a function of detuning ϵ_0, where $A = (1\mu eV)^2$ and $\partial t_0/\partial E_b = 6.4 \times 10^{-3}$. The dots are the experimental data for $J/(2\hbar)$ (circle) and $1/T_\varphi$ (square) [5]. Left: $1/T_\varphi$ due to uncorrelated noise; Right: $1/T_\varphi$ due to fully correlated noise.

rate increases with decreasing $U - \epsilon_0$. The decay can be fit to $e^{-(t/T_\varphi)^\beta}$, and we find that $\beta \approx 1.90$ for all these curves. As shown in the previous section, the decay dynamics has $e^{-A_{eff}t^2 \ln(1/(\omega_0 t))}$ dependence, where the logarithmic function leads to a slower time dependence, i.e. $\beta \lesssim 2$.

S5. NUMBER OF TWO-QUBIT OPERATIONS

Here we study the scaling of the figure of merit $N_{CZ} = JT_2^*/(2\hbar)$, effectively the number of controlled-Z (CZ) operations in a coherence time, with the detuning ϵ_0 (use $\beta = 2$), where $1/T_2^* = (1/T_\varphi + \Gamma_0)$ and Γ_0 is assumed to be the single-qubit decoherence. If detuning noise dominates the spin decoherence, $N_{CZ} = [J/(2\hbar)]/[\Gamma_1(\frac{t_0}{U-\epsilon_0})^2 + \Gamma_0]$, where $J \approx 2t_0^2/(U - \epsilon_0)$ is the exchange interaction, and Γ_1 is the two-qubit dephasing rate $1/T_\varphi(\epsilon_0 = U - t_0)$ near the charge transition point (this is the same order of dephasing as for the (1,1)-(2,0) charge qubit). Thus, the maximum of N_{CZ} happens at $\epsilon_0/U = 1 - t_0/U\sqrt{\Gamma_1/\Gamma_0}$. If $\Gamma_0 \lesssim \Gamma_1(t_0/U)^2$, then, $\epsilon_0 \to 0$ is preferred. However, if $\Gamma_0 \gg \Gamma_1(t_0/U)^2$, then, $\epsilon_0 \approx U$ is preferred for larger N_{CZ}. On the other hand, if tunneling noise dominates spin decoherence, $N_{CZ} = [J/(2\hbar)]/(\Gamma_1' \frac{t_0}{U-\epsilon_0} + \Gamma_0)$, where Γ_1' is the corresponding two-qubit dephasing rate at $\epsilon_0 = U - t_0$ near the charge transition. Thus, N_{CZ} always increases as ϵ_0 approaches U (more asymmetry) in the presence of a finite Γ_0.

Figure S4 shows the figure of merit $N_{CZ} = JT_2^*/(2\hbar)$ of CZ operations for detuning noise only (left panel) and tunneling noise only (right panel), where single-qubit decoherence rate Γ_0 is assumed in the calculation. For detuning noise, when single qubit dephasing is slow ($\Gamma_0 < 1$ kHz) , N_{CZ} increases as ϵ_0 moves away from U (more symmetric); When Γ_0 is large, there is an optimal detuning point. For tunneling noise, when $\Gamma_0 = 0$, N_{CZ} decreases slowly with increasing ϵ_0 due to the $1/(U-\epsilon_0)^{2/\beta}$ scaling, where β is slightly less than 2; When $\Gamma_0 > 1$ kHz, N_{CZ} increases with increasing ϵ_0 (more asymmetry) in most of the detuning regime because of the weaker dependence of $1/T_\varphi$ with $1/(U-\epsilon_0)$ compare to the case of detuning noise.

S6. EFFECT OF CORRELATION OF THE NOISES

Figure S5 shows a log-log plot of $J/(2\hbar)$ and dephasing rate $1/T_\varphi$ as a function of the detuning ϵ_0. The calculated dephasing rate $1/T_\varphi$ in the left (right) panel is due to

the uncorrelated (fully correlated) noise. The dots in the figures are the experimental data extracted from Ref. [5]. For correlated noises, the results are modified near the transition point where the effect of tunneling noise and detuning noise are comparable. The figure shows that the result of the uncorrelated noise matches better with the experimental data compare to the fully correlated noise.

[1] P. W. Anderson, B. I. Halperin, and C. M. Varma, Philosophical Magazine **25**, 1 (1972).
[2] W. A. Phillips, J Low Temp Phys **7**, 351 (1972).
[3] T. Thorbeck and N. M. Zimmerman, J. Appl. Phys. **111**, 064309 (2012).
[4] M. Russ and G. Burkard, Phys. Rev. B **91**, 235411 (2015).
[5] M. Veldhorst, C. H. Yang, J. C. C. Hwang, W. Huang, J. P. Dehollain, J. T. Muhonen, S. Simmons, A. Laucht, F. E. Hudson, K. M. Itoh, et al., Nature **526**, 410 (2015).
